# E3 ubiquitination ligase XIAP lightens diabetes‐induced cognitive impairment by inactivating TXNIP‐ERS‐mediated neuronal injury

**DOI:** 10.1002/kjm2.12913

**Published:** 2024-12-04

**Authors:** Qin Zhang, Hai‐Jin Huang, Jing‐Ling Zhang, Ying Tian, Ying Hu

**Affiliations:** ^1^ Department of Anesthesiology and Operative Medicine, Medical Center of Anesthesiology and Pain, the 1st Affiliated Hospital, Jiangxi Medical College Nanchang University Nanchang Jiangxi Province PR China; ^2^ Department of Endocrinology and Metabolism, the 1st Affiliated Hospital, Jiangxi Medical College Nanchang University Nanchang Jiangxi Province PR China; ^3^ Jiangxi Clinical Research Center for Endocrine and Metabolic Disease Nanchang Jiangxi Province PR China; ^4^ Jiangxi Branch of National Clinical Research Center for Metabolic Disease Nanchang Jiangxi Province PR China

**Keywords:** diabetes‐induced cognitive dysfunction, endoplasmic reticulum stress, TXNIP, XIAP

## Abstract

Diabetes‐induced cognitive dysfunction (DCD) is a neurological disorder associated with diabetes, characterized by cognitive impairment driven by neuronal injury from chronic high glucose (HG) exposure. This study aims to elucidate the role and mechanisms of the X‐linked inhibitor of apoptosis protein (XIAP)/thioredoxin‐interacting protein (TXNIP) in hippocampal neuron cell death and cognitive function within DCD models. A diabetic rat model was established using a high‐fat/sucrose diet and streptozotocin injection. Primary hippocampal neurons were stimulated with HG to mimic diabetic conditions. Cognitive and memory functions were assessed using the Morris water maze (MWM) and novel object recognition test (ORT).

## 1 INTRODUCTION

Diabetes mellitus (DM) and its complications have become a significant global health burden, with projections suggesting that it will affect 693 million adults worldwide by 2045.^1^ DM induces both microvascular and macrovascular complications, such as diabetic nephropathy, retinopathy, and neuropathy, which increase mortality and decrease the life quality of patients.^2^ Among these complications, cognitive dysfunction is recognized as a critical comorbidity. Diabetes‐induced cognitive dysfunction (DCD) encompasses cognitive impairment and dementia, profoundly affecting patients' daily life and survival.^3^, ^4^ The pathogenesis of DCD remains elusive, and abnormal neuron cell injury or death (induced by excessive apoptosis, pyroptosis, ferroptosis, and inflammation) can be the core of its potential pathophysiology.^5^, ^6^ Understanding the molecular mechanisms underlying neuronal injury and death in DCD is essential for identifying potential therapeutic targets and developing effective treatments.

The endoplasmic reticulum (ER) is a crucial organelle responsible for protein synthesis, folding, and modification, all of which are vital for cell function and survival.^7^ However, the accumulation of misfolded or unfolded proteins can disrupt ER homeostasis, triggering a condition known as endoplasmic reticulum stress (ERS).^8^ Activated ERS is closely linked to the progression of diabetes and DCD. Elevated levels of ERS markers—such as C/EBP homologous protein (CHOP), activating transcription factor 6 (ATF6), and immunoglobulin heavy‐chain‐binding protein (GRP78)—have been observed in DCD models.^9^ Persistent ERS can promote neuronal death, thereby contributing to cognitive dysfunction.^10^ For example, Zhang et al.^11^ reported that CHOP overexpression exacerbated neuronal injury and cognitive impairment in diabetic animals by intensifying ERS‐related apoptosis. Similarly, Ding et al.^12^ demonstrated that ERS activation, together with oxidative stress, triggered NLRP3 inflammasome formation and neuronal pyroptosis in a rat model of cerebral venous sinus thrombosis. These findings underscore the importance of targeting ERS to mitigate neuronal death and cognitive dysfunction in DCD.

Thioredoxin‐interacting protein (TXNIP), also known as thioredoxin‐binding protein 2, belongs to the α‐arrestin protein family and is regulated by various cellular stressors.^13^ TXNIP inhibits the antioxidant activity of thioredoxin, and can interacts with the NLRP3 inflammasome to drive inflammatory responses and pyroptosis.^14^ Increasing evidence suggests that TXNIP plays a key role in the development of DM and its complications, and targeting TXNIP could offer therapeutic benefits.^15^ Additionally, TXNIP has been implicated in cognitive dysfunction across a range of conditions, including sepsis‐induced brain injury and Alzheimer's disease.^16^, ^17^ In diabetic models, compounds such as neferine and ChemR23 have improved cognitive function by suppressing NLRP3 inflammasome activation, oxidative stress, and ERS via TXNIP inhibition.^9^, ^18^ In our previous study, we demonstrated that resveratrol alleviated hippocampal neuronal apoptosis, inflammation, and ERS by negatively regulating TXNIP, leading to improved cognitive function in DCD models.^19^ However, the precise role of TXNIP in neuronal injury and DCD progression requires further investigation.

The X‐linked inhibitor of apoptosis protein (XIAP) is a crucial regulator of cell survival, modulating processes such as apoptosis, autophagy, and necroptosis.^20^ XIAP suppresses apoptosis and inhibits inflammation‐driven cell death.^21^ In animal models, XIAP silencing exacerbates neuronal apoptosis in hypoxia–ischemia‐induced brain injury,^22^ while its overexpression promotes neuronal survival, highlighting its neuroprotective potential.^23^ XIAP has also been implicated in DM, where its overexpression reduces islet allograft rejection in diabetic patients.^24^, ^25^ As an E3 ubiquitin ligase, XIAP facilitates the ubiquitination of target proteins, thereby regulating various cellular processes.^26^ Using the UbiBrowser 2.0 database, we predicted that XIAP may promote the ubiquitination of TXNIP. Based on this, we hypothesize that XIAP ameliorates HG‐induced ERS, apoptosis, and pyroptosis by degrading TXNIP through ubiquitination, thereby improving cognitive function in DCD. Elucidating this mechanism will enhance our understanding of DCD pathophysiology and provide new therapeutic targets for its treatment.

## 2 MATERIALS AND METHODS

### 2.1 Establishment of diabetic rat models

Sprague–Dawley male rats (6 weeks old, 200 ± 12 g) were obtained from HFK Biotech (Beijing, China). The rats were divided into two groups: the sham group (*n* = 10), which received a normal diet, and the DM group (*n* = 30). The DM model, induced through a high‐fat/sucrose diet, has limitations, including variability in blood glucose levels and low induction rates with streptozotocin (STZ). To enhance model accuracy, rats were fed a high‐fat/sucrose diet (4 weeks) to induce insulin resistance before receiving an intraperitoneal (i.p.) injection of STZ (HY‐13753, 0.45%, 35 mg/kg, MCE, Shanghai, China). Blood glucose levels were measured 72 h post‐STZ injection, with a glucose level ≥ 16.7 mmol/L considered successful model induction. Lentivirus infections were performed under anesthesia with sodium pentobarbital (i.p., 40 mg/kg). Bilateral injections of lentivirus‐XIAP and/or lv‐TXNIP (1 × 10^9^ transducing units/mL) into the hippocampus were conducted over 14 days. At the end of the experiment, half of the rats were euthanized to collect blood and brain tissue samples, while the others were evaluated for neurocognitive function. All animal experiments were approved by the Research Ethics Committee of the author's hospital (CDYFY‐IACUC‐202301QR032).

### 2.2 Morris water maze test

The Morris water maze (MWM) test was used to assess spatial learning and memory in rats. The trials took place in a circular pool (180 cm diameter, 50 cm height) with four starting points located along the wall. During the acquisition trials, the rats were trained to locate a hidden platform within 1 min. If a rat failed to find the platform within this time, it was guided to the platform and allowed to remain there for 15 s. Each rat underwent training for four consecutive days, with time spent searching for the platform recorded. On the fifth day, the platform was removed for the probe trial to assess memory retention. Rats were placed at new starting locations, and the time spent in the target quadrant was recorded over 1 min. The Smart digital tracking & analysis system (Panlab, Barcelona, Spain) was used to analyze the results.

### 2.3 Novel object recognition test

The object recognition test (ORT) was conducted following our previously described procedures.^19^ To reduce stress, the rats were stroked for 1–2 min daily before testing. During the inhabitation stage, each rat explored a novel space for 5 min. After a 24‐h interval, the training stage involved 5 min of exploration in an arena containing two identical objects. On the test day, one object was replaced with a novel object. The rat's behavior was observed for 5 min, and the discrimination index was calculated using the formula: discrimination index = time spent with the new object/(time spent with the older object + time spent with the new object) × 100.

### 2.4 Hematoxylin–eosin staining

Brain tissue samples collected from rats were fixed with 4% paraformaldehyde, dehydrated, and embedded in paraffin. Sections (5 μm thickness) were prepared using a HistoCore AUTOCUT microtome (Leica Biosystems, Shanghai, China). The sections were stained with hematoxylin and eosin at room temperature to visualize pathological changes under a microscope (200× magnification, DM2500, Leica Biosystems).

### 2.5 Terminal deoxynucleotidyl transferase‐mediated dUTP nick‐end labeling assays

Terminal deoxynucleotidyl transferase‐mediated dUTP nick‐end labeling (TUNEL) assays were performed to assess apoptosis in brain tissues collected from the rats. Samples were embedded in optimal cutting temperature (OCT) media (Tissue‐Tek, Tokyo, Japan) and sectioned to 8 μm thickness. The TUNEL assay kit (#C1091, Beyotime Biotech, Shanghai, China) was used according to the manufacturer's protocol. Briefly, sections were incubated sequentially with 0.3% Triton X‐100 (5 min, 25°C), 0.3% H₂O₂ (20 min, 25°C), biotin diluent (60 min, 37°C), streptavidin–HRP (30 min, 25°C), and DAB solution (10 min, 25°C). Apoptotic cells were observed under a microscope (200× magnification, Leica Biosystems), and the apoptosis ratio was calculated as the percentage of TUNEL‐positive cells relative to total cells in the control group.

### 2.6 Rat hippocampal neuron isolation

Primary hippocampal neurons were isolated following previously established methods.^19^ Briefly, embryos from pregnant female rats were collected, and their heads were placed in ice‐cold phosphate‐buffered saline (PBS). Using a Leica stereomicroscope (Leica Biosystems), the hippocampus was carefully dissected. The isolated tissue was digested in papain buffer for 10 min at 37°C and centrifuged for 5 min using a Micro17R refrigerated centrifuge (Thermo Scientific, Shanghai, China). Afterwards, the obtained cell pellet was resuspended and maintained as primary hippocampal neurons in a neurobasal growing medium.

### 2.7 Cell culture and treatment

The cell culture procedure was carried out in line with our previous study.^19^ The isolated primary hippocampal neurons were maintained in a neurobasal medium (every 3 days replacing the half‐medium to fresh medium) and were cultivated in humidified incubator. To simulate diabetic conditions, neurons were exposed to high glucose (HG, 50 mmol/L) for 48 h. To avoid the effect of high osmotic factors (OS), mannitol (MA, 50 mmol/L) was utilized to treat hippocampal neuron cells as the OS group.

### 2.8 Lentivirus vector infection

Lentivirus packaging plasmids for XIAP and TXNIP, along with negative controls, were obtained from GenePharma (Shanghai, China). Primary hippocampal neurons (10^5^ cells per well) were seeded in six‐well plates with serum‐free medium. Lentiviruses were applied at a multiplicity of infection (MOI) of 30. After 4 h of incubation, the medium was replaced with complete medium for an additional 48 h. The overexpression efficiency was detected by qRT‐PCR assay.

### 2.9 Cell viability assays

After the corresponding treatment for primary hippocampal neuron cells, the cell viabilities were evaluated employing the cell counting kit (CCK‐8, #40203ES60, Yeasen Biotech, Shanghai, China). Cells were cultured in 96‐well plates at a density of 3 × 10^4^ cells per well for 24 h. Subsequently, the cells were incubated for an additional 2 h, after which 10 μL of CCK‐8 solution was supplemented. Following this, the absorbance at 450 nm was measured using a microplate reader (Molecular Devices, San Jose, CA, USA).

### 2.10 Enzyme‐linked immunosorbent assay

The levels of interleukin (IL)‐1β (#ab214025, Abcam, Cambridge, UK) and IL‐18 (#ab215539, Abcam) levels in brain tissue homogenates and hippocampal neuron supernatants were measured using enzyme‐linked immunosorbent assay (ELISA) kits following the manufacturer's guidelines. Absorbance at 450 nm was recorded with a microplate reader (Molecular Devices) to determine cytokine concentrations.

### 2.11 Co‐immunoprecipitation analysis

To verify the interaction between XIAP and TXNIP, hippocampal neuron cells (2 × 10^5^) were lysed, and the lysate was incubated with anti‐TXNIP antibody (1 μg, #18243‐1‐AP, Proteintech, Rosemont, IL, USA) or control IgG (1 μg, #30000‐0‐AP, Proteintech) for 12 h, at 4°C. The antigen–antibody complexes were mixed with protein A/G beads (#88803, Thermo Scientific, Shanghai, China) and incubated for 3 h following the manufacturer's instructions. The complexes were eluted and analyzed by Western blotting to assess XIAP and TXNIP abundance.

### 2.12 Flow cytometry analysis

Apoptosis and pyroptosis were assessed using the Annexin V‐FITC Apoptosis Detection Kit (#abs50001, Absin Biotech, Shanghai, China) and the Active Caspase‐1 Detection Kit (ImmunoChemistry, Bloomington, MN, USA). Hippocampal neurons (1 × 10^6^ cells/well) were resuspended in 100 μL of 1× binding buffer, stained with V‐FITC (5 μL) or FLICA 660‐YVAD‐FMK (5 μL) for 10 min, and then incubated with propidium iodide (PI, 5 μL) in the dark for 5 min. Subsequently, the cell suspension was supplemented with an additional 1 × binding buffer (400 μL) for the assessment of apoptotic or pyroptotic cells using the Attune NxT flow cytometer (Thermo Fisher, Waltham, MA, USA). Cells with Annexin V^+^ PI^−^ were identified as early apoptotic cells, while Annexin V^+^ PI^+^ cells indicated late apoptosis. Caspase‐1^+^ PI^+^ cells were considered pyroptotic. Each experiment was performed three times independently.

### 2.13 Quantitative real‐time polymerase chain reaction

Total RNA from brain tissues and primary hippocampal neurons was extracted using TRIeasy™ LS Total RNA Extraction Reagent (#19201ES60, Yeasen Biotech, Shanghai, China). Subsequently, cDNA synthesis was performed using the Quantscript RT Kit (#KR103, Tiangen, Beijing, China). Relative mRNA levels were determined with the Hieff qPCR SYBR Green Master Mix (No Rox, #11201ES08, Yeasen Biotech). GAPDH served as the internal control, and the 2^−∆∆Ct^ method was used for data analysis. Primer sequences are listed in Table S1.

### 2.14 Western blotting analysis

Total protein extraction was performed using RIPA lysis buffer (#20101ES60, Yeasen Biotech) and quantified with a BCA Protein Assay Kit (#PC0020, Solarbio Biotech, Beijing, China). Protein samples (15 μg) were resolved by 15% SDS–PAGE and transferred to polyvinylidene difluoride (PVDF) membranes (Millipore, Billerica, MA, USA) at 4°C. Membranes were blocked with 5% bovine serum albumin for 1 h at 25°C, followed by incubation with primary antibodies for 12 h at 4°C. Secondary antibodies conjugated with horseradish peroxidase were applied for 1 h at 25°C. Protein bands were visualized using the Enhanced ECL Chemiluminescent Substrate Kit (#36222ES60, Yeasen Biotech) and the ImageQuant LAS system (GE Healthcare, Sunnyvale, CA, USA). The primary antibodies including TXNIP (1:1000, #18243‐1‐AP), BIRC3 (1:1000, #24304‐1‐AP), CBLB (1:1000, #12781‐1‐AP), RBX1 (1:1000, #14895‐1‐AP), XIAP (1:1000, #10037‐1‐Ig), and GPR78 (1:1000, #11587‐1‐AP) were obtained from Proteintech. CHOP (1:1000, #2895), ATF6 (1:1000, #65880), and β‐actin (1: 500, #93473) antibodies were purchased from CST (Beverly, MA, USA). β‐actin was selected as the control for normalization.

### 2.15 Ubiquitination determination

Total protein from brain tissues and hippocampal neurons was isolated and pretreated by boiling and dilution. Protein A/G beads (Thermo Scientific) were incubated with anti‐TXNIP (#18243‐1‐AP) or anti‐IgG (#30000‐0‐AP) antibodies. The antibody–protein complexes were incubated with samples for 12 h at 4°C, eluted, and analyzed by Western blotting. Anti‐ubiquitin antibody (#ab7780, Abcam) was used to detect ubiquitinated proteins.

### 2.16 Statistical analysis

Data analysis was performed using SPSS 20.0 software (SPSS Inc., Chicago, IL). Comparisons between two groups were made using the Student's *t*‐test, while multiple group comparisons were analyzed by one‐way ANOVA followed by Tukey's post hoc test. Results are expressed as mean ± standard deviation (SD). Each experiment was repeated at least three times. Statistical significance was defined as *p* < 0.05.

## 3 RESULTS

### 3.1 The elevated TXNIP expression in brain tissues of diabetic rats was correlated to its decrease in ubiquitination level

**FIGURE 1:**
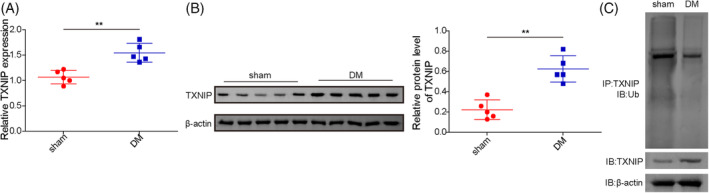
The elevated TXNIP expression in brain tissues of diabetic rats was correlated to its decrease in ubiquitination levels. SD rats were fed a high‐fat/sucrose diet for 4 weeks followed by streptozotocin injection. Brain tissues were collected for analysis. (A) The mRNA level of TXNIP was detected by qRT‐PCR analysis. (B) TXNIP protein expression was analyzed by Western blotting. (C) TXNIP ubiquitination level was assessed using ubiquitination assays. Results are expressed as mean ± standard deviation (SD). *N* = 5 per group. Statistical significance: *p < 0.05, **p < 0.01, ***p < 0.001.

The DM model was successfully established through a high‐fat/sucrose diet followed by streptozotocin injection. Compared with sham rats, the mRNA and protein level of TXNIP were significantly upregulated in the brain tissues of diabetic rats (Figure 1A,B). The ubiquitination modification levels of TXNIP in the brain tissues of DM rats were down‐regulated than the sham group (Figure 1C). Collectively, these data indicated that the abnormally expressed TXNIP protein in DCD rats was related to its protein ubiquitination modification.

### 3.2 E3 ubiquitination ligase XIAP was downregulated in DCD and involved in the ubiquitination regulation of TXNIP protein

**FIGURE 2:**
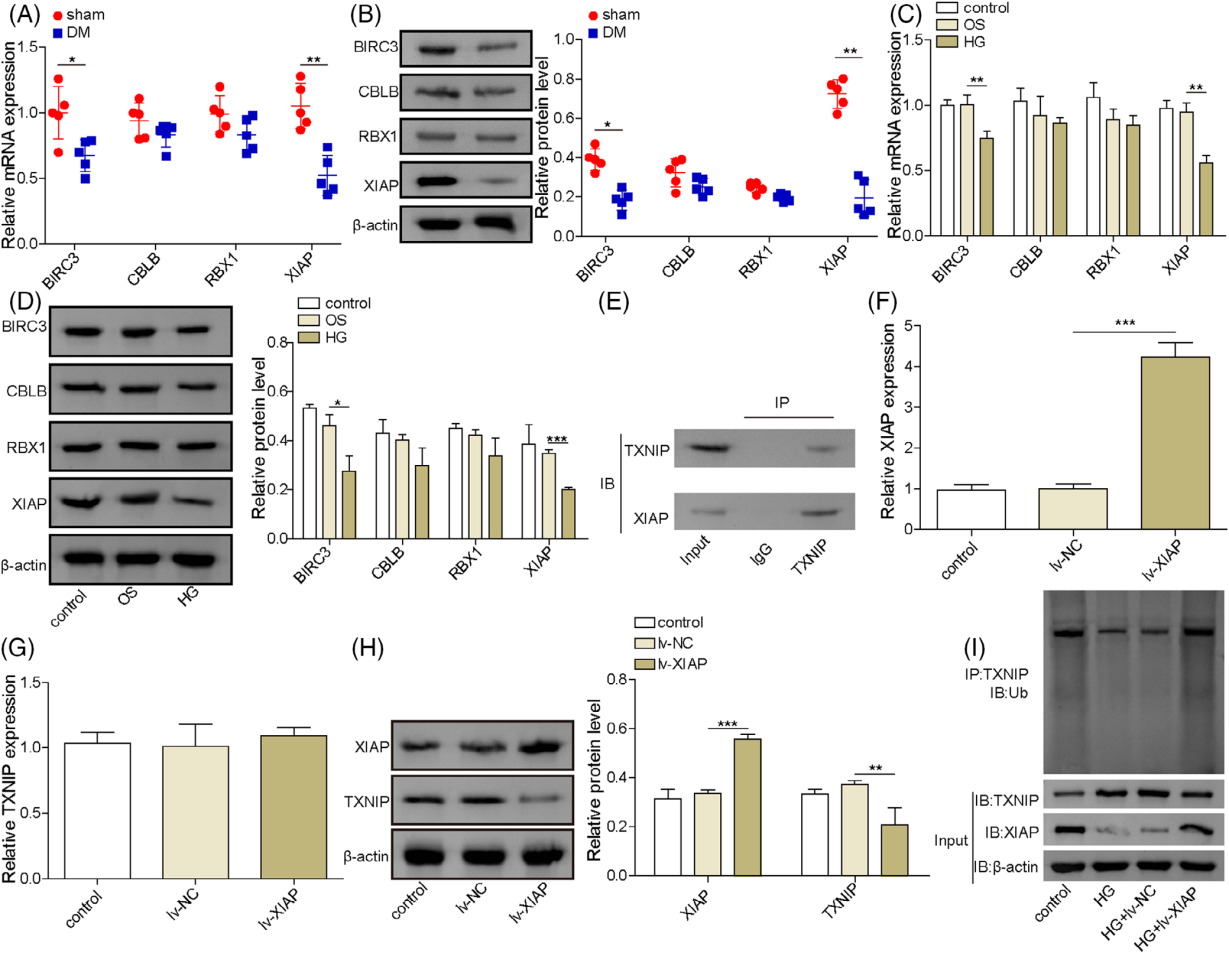
E3 ubiquitination ligase XIAP was involved in the ubiquitination regulation of TXNIP protein. (A) The mRNA levels of BIRC3, CBLB, RBX1, and XIAP were measured in brain tissues from rats in the sham or DM group by qRT‐PCR. (B) The protein levels of BIRC3, CBLB, RBX1, and XIAP in the brain tissues from rats in the sham or DM group were measured by Western blotting. (C, D) The mRNA and protein levels of BIRC3, CBLB, RBX1, and XIAP in HG‐treated hippocampal neurons were analyzed by qRT‐PCR and Western blotting, respectively. (E) Co‐IP analysis confirmed the interaction between XIAP and TXNIP in hippocampal neurons. (F, G) qRT‐PCR detected the mRNA levels of XIAP and TXNIP in hippocampal neurons with lv‐XIAP or lv‐NC infection. (H) Western blotting examined the protein expressions of XIAP and TXNIP in hippocampal neurons with lv‐XIAP or lv‐NC infection. (I) Ubiquitination assays determined the TXNIP ubiquitination levels. Animal experiments were repeated five times, and cellular experiments were performed in triplicate. Results are expressed as mean ± SD. Statistical significance: *p < 0.05, **p < 0.01, ***p < 0.001.

Using the UbiBrowser 2.0 database, several potential E3 ubiquitin ligases for TXNIP, including BIRC3, CBLB, RBX1, and XIAP, and its and E3 recognizing motif were identified (Figures S1 and S2). There were no significant changes in the expression of CBLB and RBX1 between DM and sham rats (Figure 2A,B). However, the mRNA and protein levels of BIRC3 and XIAP were significantly down‐regulated in the DM rats' brain tissues, compared with the sham rats, especially XIAP. Next, we isolated the rat. To further explore XIAP's role, primary hippocampal neurons were isolated, and a high‐glucose (HG)‐induced cell model was developed. Consistent with in vivo findings, HG exposure reduced the mRNA and protein levels of XIAP and BIRC3, while CBLB and RBX1 remained unchanged (Figure 2C,D). Co‐immunoprecipitation analysis confirmed a direct interaction between XIAP and TXNIP, as XIAP was enriched in the anti‐TXNIP immunoprecipitate compared to the IgG control (Figure 2E). Overexpression of XIAP by lentiviral transduction significantly increased XIAP mRNA expression without affecting TXNIP mRNA levels (Figure 2F,G). However, XIAP overexpression enhanced its protein expression and suppressed TXNIP protein level in hippocampal neurons (Figure 2H). Additionally, XIAP overexpression rescued the downregulated TXNIP ubiquitination levels caused by HG exposure (Figure 2I). These results demonstrated that XIAP play a critical role in regulating TXNIP ubiquitination.

### 3.3 Overexpression of XIAP alleviated HG‐induced ERS and apoptosis in hippocampal neuron cells by inhibiting TXNIP

**FIGURE 3:**
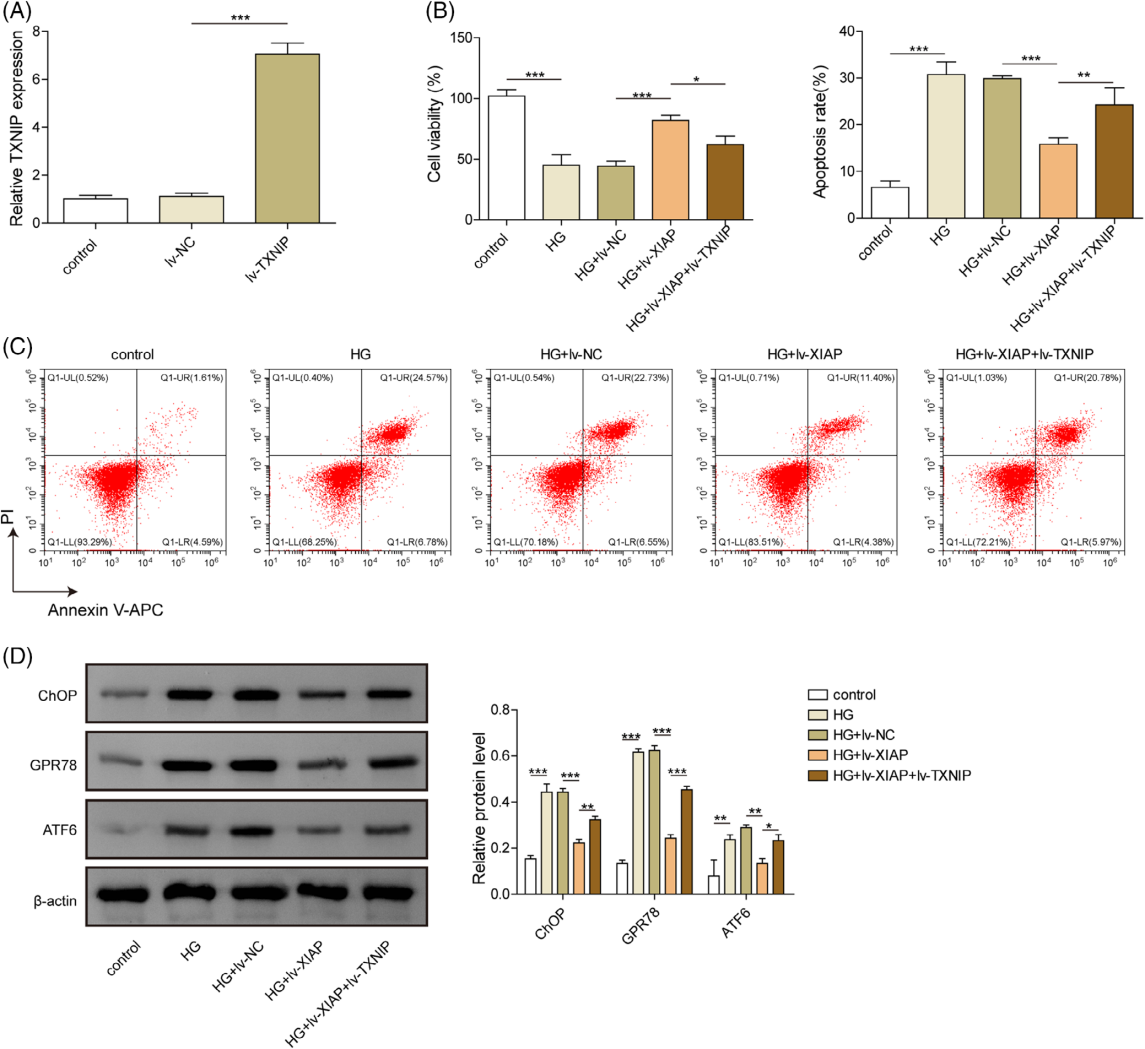
Overexpression of XIAP alleviated HG‐induced ERS and apoptosis in hippocampal neuron cells by inhibiting TXNIP. (A) TXNIP mRNA level was measured by qRT‐PCR following transfection with lv‐NC or lv‐TXNIP. (B) Neuronal viability was assessed using the CCK‐8 assay. (C) Apoptosis was measured by flow cytometry. (D) ERS‐associated proteins (CHOP, ATF6, and GPR78) were analyzed by Western blotting. Each experiment was performed in triplicate. Results are presented as mean ± SD, *n* = 3 per group. Statistical significance: *p < 0.05, **p < 0.01, ***p < 0.001.

To investigate the functional role of XIAP/TXNIP, hippocampal neurons were transduced with lv‐XIAP alone or co‐infected with lv‐TXNIP. Compared with the lv‐NC group, TXNIP mRNA level was significantly increased in neurons co‐infected with lv‐TXNIP (Figure 3A). XIAP overexpression significantly enhanced cell viability in HG‐treated neurons, but this protective effect was abolished by TXNIP co‐overexpression (Figure 3B). XIAP overexpression also reduced apoptosis in HG‐treated neurons, while TXNIP co‐overexpression reversed this anti‐apoptotic effect (Figure 3C). Furthermore, XIAP overexpression markedly decreased the expression of ERS markers (CHOP, ATF6, and GPR78), but these effects were negated by TXNIP co‐overexpression (Figure 3D). Together, these results indicated that XIAP mitigated HG‐induced ERS and apoptosis by inhibiting TXNIP.

### 3.4 XIAP inhibited HG‐induced NLRR3 inflammasome and pyroptosis in hippocampal neurons via repressing TXNIP

**FIGURE 4:**
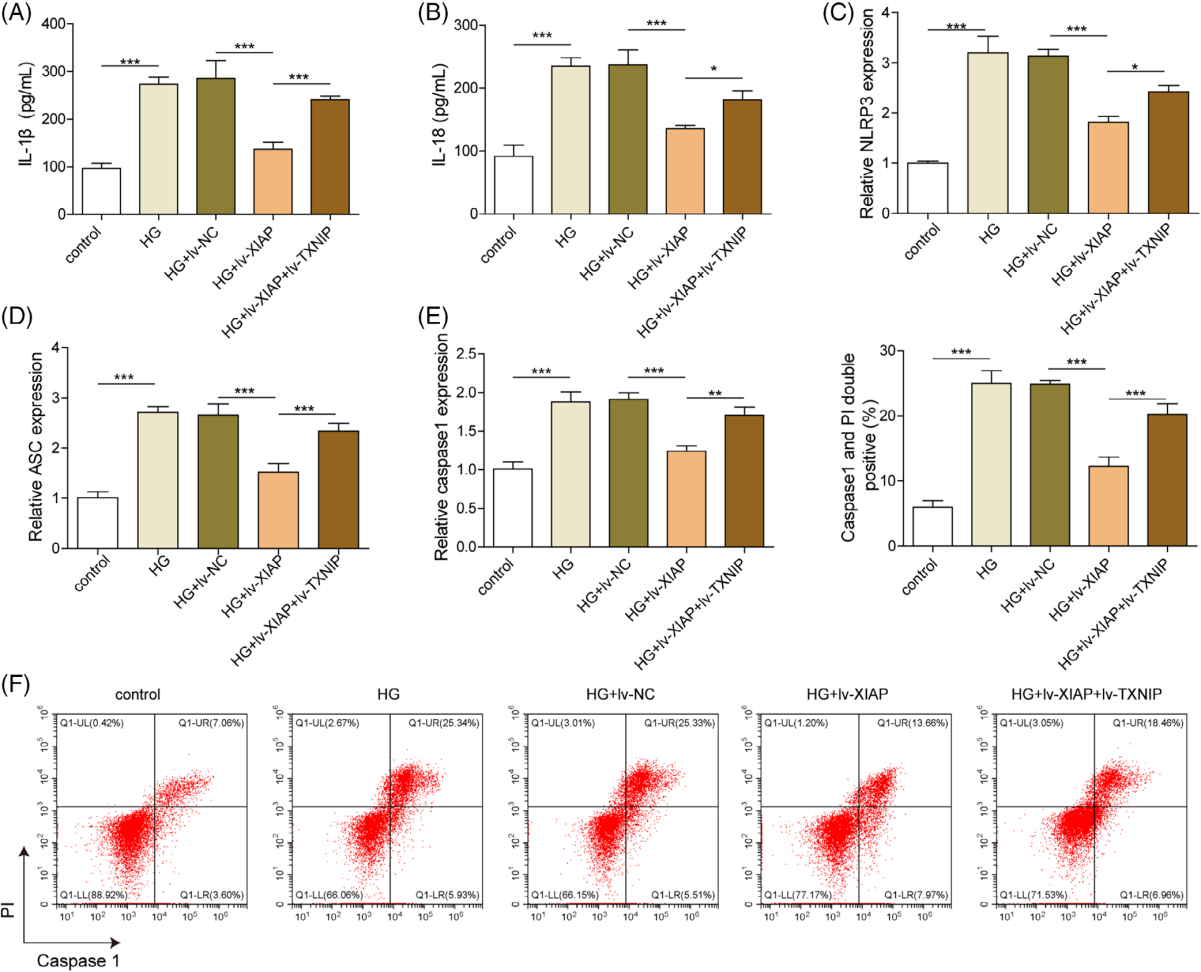
XIAP inhibited HG‐induced NLRR3 inflammasome and pyroptosis in hippocampal neurons via repressing TXNIP. Primary hippocampal neurons were stimulated with HG and infected with the lv‐NC, lv‐XIAP, lv‐XIAP + TXNIP vectors. (A) ELISA kits evaluated the IL‐1β level. (B) ELISA kits evaluated the IL‐18 level. (C) qRT‐PCR detected the mRNA level of NLRP3. (D) qRT‐PCR detected the mRNA level of ASC. (E) qRT‐PCR detected the mRNA level of Caspase 1. (F) Flow cytometry measured the cell pyroptosis. Each experiment was performed in triplicate. Results are expressed as mean ± SD, *n* = 3 per group. Statistical significance: *p < 0.05, **p < 0.01, ***p < 0.001.

The role of XIAP/TXNIP in HG‐induced pyroptosis was further evaluated. HG exposure significantly increased the secretion of pro‐inflammatory cytokines IL‐1β and IL‐18, while XIAP overexpression reduced their levels (Figure 4A). However, this anti‐inflammatory effect was weakened by TXNIP co‐overexpression (Figure 4A). Similarly, the expression of NLRP3 inflammasome‐related genes (NLRP3, ASC, and Caspase‐1) was decreased by XIAP overexpression, but these effects were reversed by TXNIP co‐overexpression (Figure 4B). Pyroptosis, which was elevated under HG conditions, was reduced by XIAP overexpression but further enhanced by TXNIP co‐overexpression (Figure 4C). These findings suggested that XIAP overexpression alleviated the HG‐induced NLRP3 inflammasome and pyroptosis by TXNIP inhibition.

### 3.5 Elevation of XIAP lightened diabetic‐induced neuron injury

**FIGURE 5:**
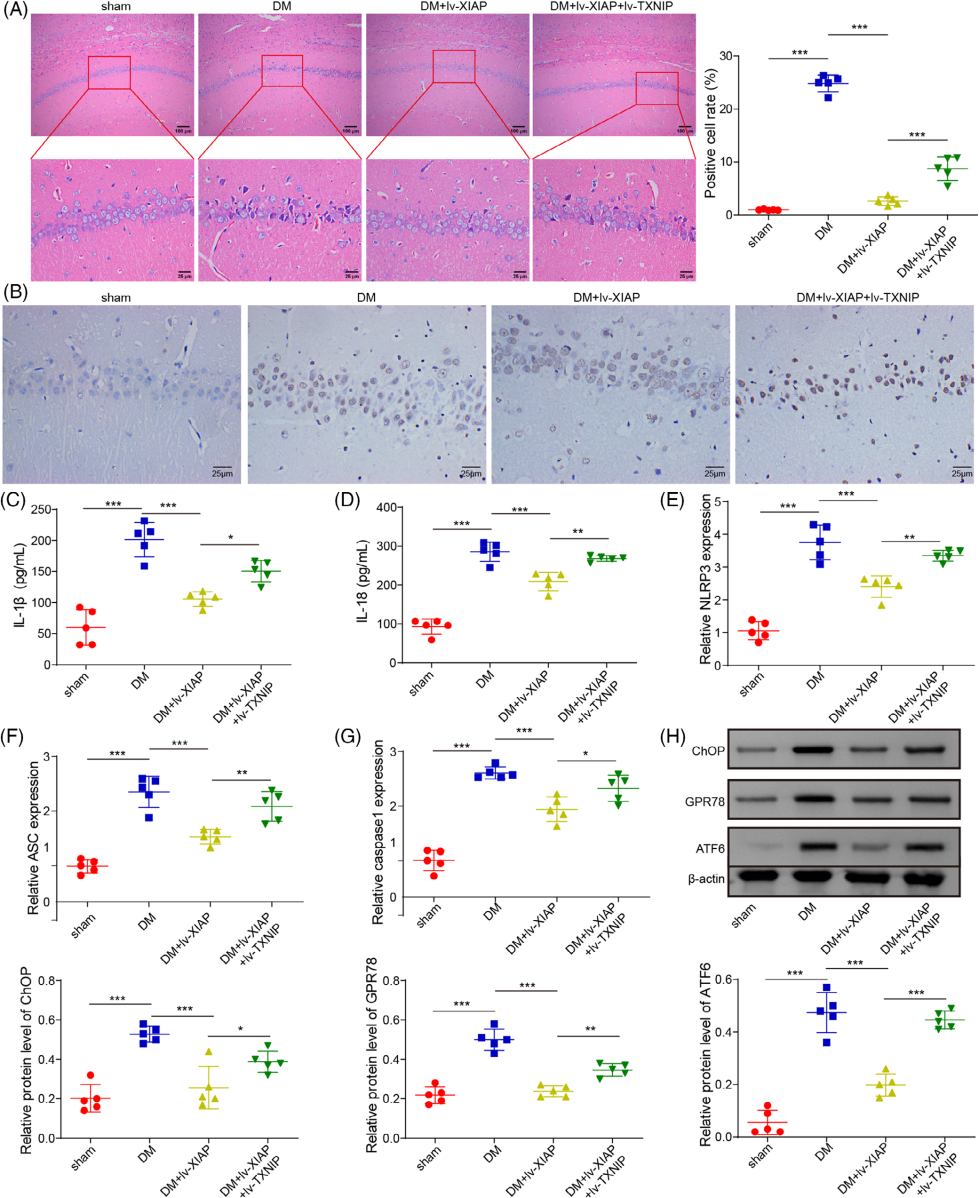
Elevation of XIAP lightened diabetic‐induced nerve injury. SD rats were fed a high‐fat/sucrose diet for 4 weeks, injected with streptozotocin (i.p.), and then infected with the lv‐NC, lv‐XIAP, lv‐XIAP + TXNIP vectors. (A) HE staining observed the histopathological changes in the hippocampal tissues of rats. (B) TUNEL staining assessed the cell apoptosis. (C, D) ELISA kits evaluated the IL‐1β and IL‐18 levels. (E–G) qRT‐PCR detected the mRNA levels of NLRP3, ASC, and Caspase 1. (H) Western blotting examined the protein levels of CHOP, ATF6, and GPR78. To ensure statistical robustness, each experiment was replicated thrice. Each experiment was conducted in triplicate. Results are shown as mean ± SD, *n* = 5 per group. Statistical significance: *p < 0.05, **p < 0.01, ***p < 0.001.

To further validate the function of XIAP/TXNIP in the DCD rat model, the lentivirus particles encoding XIAP or TXNIP were injected into the hippocampus of DM rats. HE staining revealed that XIAP overexpression significantly reduced neuronal damage, including shrunken cells, nuclear pyknosis, chromatin aggregation, and cytoplasmic reduction. However, these protective effects were diminished by TXNIP co‐overexpression (Figure 5A). TUNEL staining confirmed reduced neuronal apoptosis in the XIAP‐overexpressing group, but this effect was weakened by TXNIP co‐overexpression (Figure 5B). Similarly, XIAP overexpression lowered IL‐1β and IL‐18 levels in hippocampal tissues, but TXNIP co‐overexpression reversed these effects (Figure 5C). Consistently, the expression of NLRP3, ASC, and Caspase‐1 was downregulated by XIAP overexpression, but these changes were overturned by TXNIP co‐overexpression (Figure 5D). XIAP also suppressed ERS‐related protein expression (CHOP, ATF6, and GPR78), but this suppression was negated by TXNIP co‐overexpression (Figure 5E). These results demonstrated that XIAP alleviated diabetic‐induced neuronal injury through TXNIP inhibition.

### 3.6 XIAP ameliorated diabetic‐induced cognitive dysfunction by negatively modulating TXNIP

**FIGURE 6:**
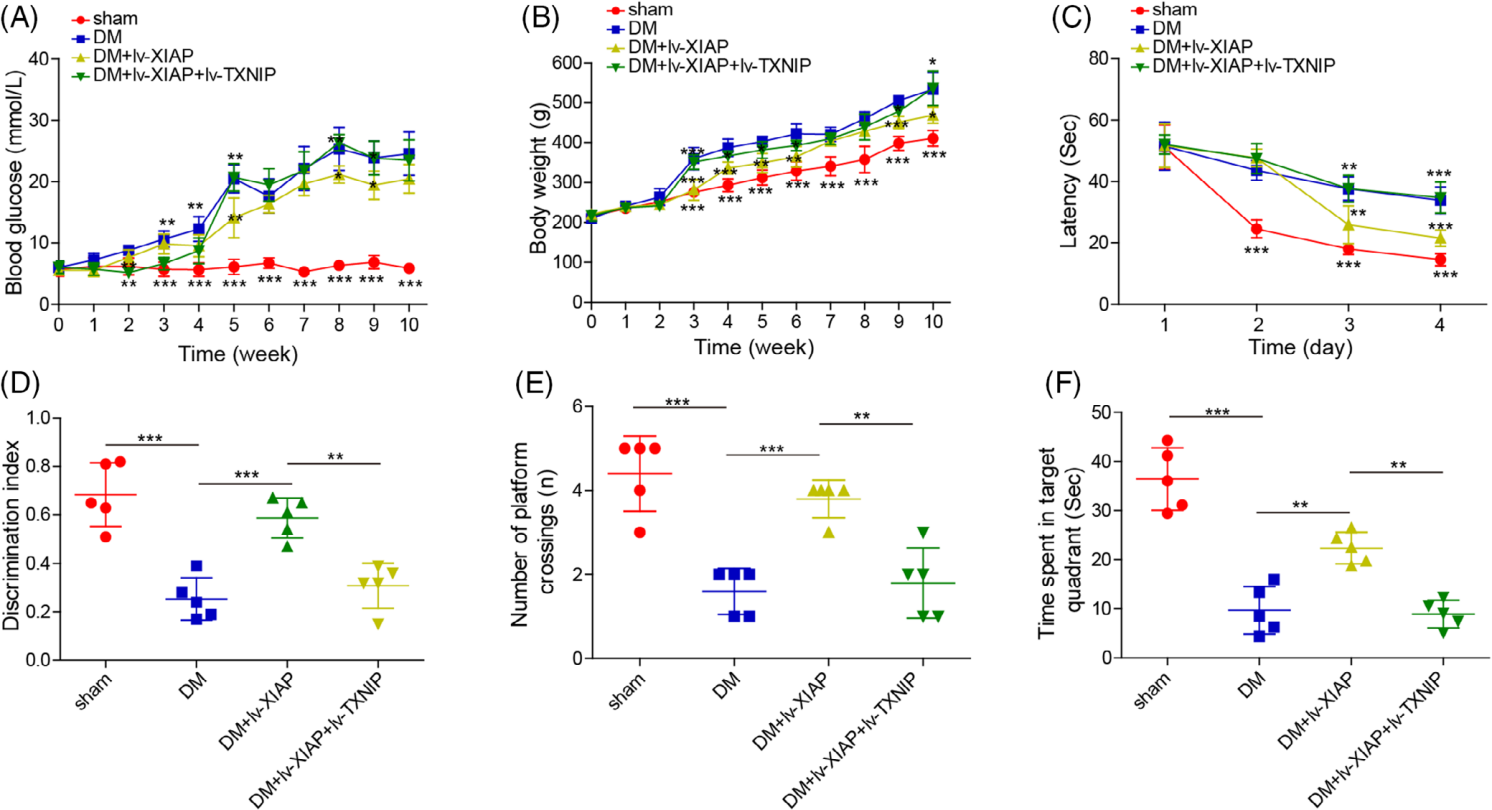
XIAP ameliorated diabetic‐induced cognitive dysfunction by negatively modulating TXNIP. SD rats were fed a high‐fat/sucrose diet for 4 weeks, injected with streptozotocin (i.p.), and then infected with the lv‐NC, lv‐XIAP, lv‐XIAP + TXNIP vectors. (A) Blood glucose was determined. (B) Body weight was recorded using an electronic balance. (C) Escape latency of the 4‐day hidden‐platform test. (D) The discrimination index of rats in the novel object recognition test. (E) Number of targets crossing in the probe trial. (F) ORT detected the time spent in the target quadrant. To ensure statistical robustness, each experiment was replicated thrice. Each experiment was performed in triplicate. Results are presented as mean ± SD, *n* = 5 per group. Statistical significance: *p < 0.05, **p < 0.01, ***p < 0.001.

Finally, the impact of XIAP on cognitive function in DM rats was evaluated. Compared with sham rats, DM rats exhibited significantly higher blood glucose level and body weight, which were reduced by XIAP overexpression. However, these improvements were abolished by TXNIP co‐overexpression (Figure 6A,B). During the MWM test, DM rats showed prolonged escape latency and a reduced discrimination index, both of which were improved by XIAP overexpression. However, these cognitive improvements were reversed by TXNIP co‐overexpression (Figure 6C,D). In the spatial probe test, XIAP overexpression enhanced memory function, as evidenced by increased target crossings and time spent in the target quadrant, but these effects were diminished by TXNIP co‐overexpression (Figure 6E,F). In summary, these findings indicated that XIAP overexpression alleviated diabetic‐induced cognitive dysfunction by negatively regulating TXNIP, highlighting the therapeutic potential of XIAP in DCD.

## 4 DISCUSSION

The incidence of diabetes and its complications has increased significantly in recent years, contributing to high mortality rates and negatively affecting the health and quality of life of patients. Diabetic cognitive decline (DCD) is a common complication of DM, with patients being 1.5–2 times more likely to experience cognitive impairment or dementia compared to the general population, particularly among the elderly.^19^ Unfortunately, no specific treatments currently target DCD. Neuronal death plays a pivotal role in cognitive decline, and protecting hippocampal neurons could alleviate cognitive dysfunction in DCD.^4^ Excessive apoptosis and pyroptosis driven by ERS exacerbate neuronal damage. Therefore, targeting ERS‐induced neuronal injury offers a potential strategy to improve cognitive function in DCD. In our study, we identified that the X‐linked inhibitor of apoptosis protein (XIAP) mitigated cognitive dysfunction by suppressing TXNIP‐mediated ERS and neuronal injury. These findings provided insights into the pathogenesis of DCD and suggested potential therapeutic targets.

TXNIP is a stress‐responsive modulator involved in various biological and pathological processes. TXNIP induces oxidative stress, inflammation, and pyroptosis through the activation of the NLRP3 inflammasome in metabolic disorders.^14^ Elevated TXNIP expression has been reported in multiple DM models.^19^ Similarly, we found that TXNIP was upregulated in both DCD rat model and HG‐treated hippocampal neurons, consistent with our previous studies.^19^ TXNIP overexpression has been associated with increased ERS and apoptosis in diabetic neurons.^19^, ^27^, ^28^ In this study, we confirmed that elevated TXNIP level was accompanied by increased ERS markers (CHOP, ATF6, and GPR78) and apoptosis in hippocampal neurons. Additionally, TXNIP has been linked to ERS‐mediated pyroptosis in diabetic nephropathy models.^29^, ^30^ Our findings further demonstrated that TXNIP overexpression contributed to neuronal injury by activating the ERS/NLRP3 inflammasome pathway, leading to cognitive impairments in DCD. These results highlight TXNIP's central role in ERS‐mediated neuronal damage and cognitive dysfunction in DCD.

XIAP is a known modulator of cell apoptotic death and survival in miscellaneous pathological processes. As reported, XIAP has a neuroprotective function in different neuron injury models, such as hypoxia–ischemia‐induced brain injury.^22^ In our study, we first found that XIAP expression was down‐regulated in the DCD rat and neuron cell model. We also revealed that overexpression of XIAP could repress the ERS in the HG‐treated hippocampus neurons. That result was partial to the experimental evidence that inhibited ERS accompanied the up‐regulated XIAP in dopamine neurons.^31^ Our data also demonstrated that XIAP overexpression reduced HG‐induced apoptosis and promoted neuronal survival, corroborating previous study.^23^ Notably, this study was the first time to show that XIAP inhibited NLRP3 inflammasome‐mediated pyroptosis in hippocampal neurons. Additionally, XIAP overexpression alleviated memory and cognitive deficits in DCD rats, revealing its neuroprotective role in mitigating neuronal injury through the inhibition of TXNIP‐mediated ERS and pyroptosis.

Ubiquitination plays a crucial role in regulating cellular stress and survival in DM‐related complications.^32^ As a cell stress regulator, the ubiquitination enhancement of TXNIP can exert protective effects in diabetes models, such as the ischemic hind limb model of diabetic mice.^33^ Whereas, the ubiquitination modification mechanism of TXNIP, and its related modulatory effects on neuron injury and cognitive dysfunction of DCD are unclear. In our study, we initially uncovered that the TXNIP's ubiquitination modification level was down‐regulated in the brain tissues of the DCD rat. These results provided an innovative ubiquitination studying direction for TXNIP in diabetes and its complications, besides DCD. In addition, XIAP is one of the crucial ubiquitin E3 ligases that catalyze its substrate protein in ubiquitination processes, such as its downstream proteins p62 and Cdc42 in tumorigenesis.^26^, ^34^, ^35^ We conducted the prediction of the potential E3 ligases for TXNIP ubiquitination, according to the UbiBrowser 2.0 database, and we validated that XIAP E3 ligases mediated the ubiquitination modification of TXNIP in HG‐treated hippocampal neurons, for the first time. Moreover, we also identified that the XIAP overexpression‐induced TXNIP ubiquitination enhancement could alleviate cognitive dysfunction by repressing ERS‐mediated neuron injury in the DCD rat and cell models, which enriched the upstream mechanisms of TXNIP in DCD and offered more accurate regulatory targets for the potential clinical intervention of DCD.

In conclusion, the present study demonstrated that XIAP mitigated diabetic‐induced cognitive dysfunction and neuronal injury by reducing TXNIP‐ERS‐mediated apoptosis and pyroptosis. Our findings elucidated the role of the XIAP/TXNIP/ERS axis in the pathogenesis of DCD and suggested that this axis might serve as a target for therapeutic intervention of DCD. However, further research is needed to validate these findings in other models, such as the spontaneous type 2 diabetic db/db mouse model. Expanding this research will provide deeper insights into the molecular mechanisms underlying DCD and facilitate the development of targeted treatments.

## CONFLICT OF INTEREST STATEMENT

The authors declare no conflicts of interest.

## ETHICS STATEMENT

Ethical approval and implementation of all animal experiments adhered to the guidelines specified by the Research Ethics Committee of the author's hospital.

## Supporting information


FIGURE S1:

FIGURE S2:

**TABLE S1:** The primers for quantitative real‐time polymerase chain reaction assay.

## Data Availability

The data that support the findings of this study are available from the corresponding author upon reasonable request.
