# Supplementary figures and images for: E3 ubiquitination ligase XIAP lightens diabetes‐induced cognitive impairment by inactivating TXNIP‐ERS‐mediated neuronal injury
Source: Kaohsiung J Med Sci. 2024 Dec 4;41(1):e12913. doi: 10.1002/kjm2.12913 (PMC11724162; doi:10.1002/kjm2.12913)

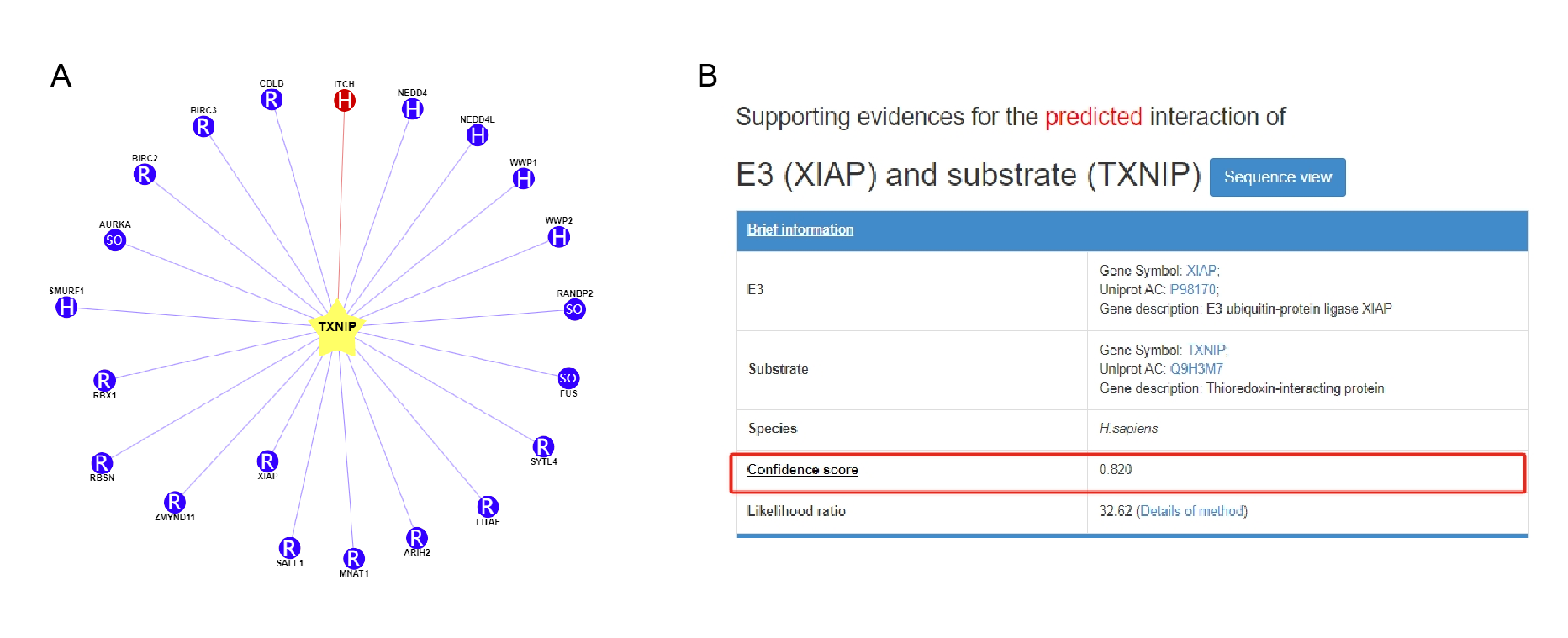

Supplement: Supplementary file 1 — FIGURE S1: [file KJM2-41-e12913-s003.tif]

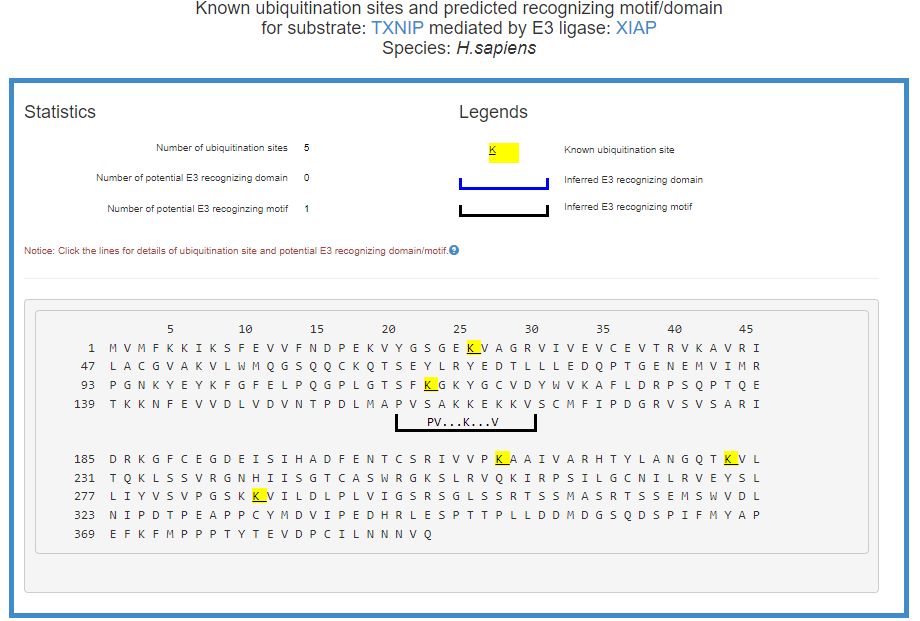

Supplement: Supplementary file 2 — FIGURE S2: [file KJM2-41-e12913-s001.tif]
