# Supplementary material for: E3 ubiquitination ligase XIAP lightens diabetes‐induced cognitive impairment by inactivating TXNIP‐ERS‐mediated neuronal injury
Source: Kaohsiung J Med Sci. 2024 Dec 4;41(1):e12913. doi: 10.1002/kjm2.12913 (PMC11724162; doi:10.1002/kjm2.12913)
Supplement: Supplementary file 3 — TABLE S1: The primers for quantitative real‐time polymerase chain reaction assay. [file KJM2-41-e12913-s002.docx]

**Supplementary Materials**

**Table 1. The primers for quantitative real-time polymerase chain reaction assay**

| Gene | Primer sequences (5’-3’) |
| --- | --- |
| TXNIP | F: GTTGCGTAGACTACTGGGTGAAG  R: CTCCTTTTTGGCAGACACTGGTG |
| XIAP | F: GACAGTATGCAAGATGACGTCAAGTCA  R: GCAAAGCTTCTCCTCTTGCAG |
| BIRC3 | F: AAGCTACCTCTCAGCCTACTTT  R: CCACTGTTTTCTGTACCCGGA |
| CBLB | F: CACCCTTCTCCCAAGCATAA  R: AGACCGAACAGGAGCTTTGA |
| RBX1 | F: CTGGCTCAAAACACGACAGG  R: AGCATCCGTTCCAGAATCCAA |
| NLRP3 | F: GATCTTCGCTGCGATCAACA  R: GGGATTCGAAACACGTGCATTA |
| ASC | F: CAGCAACACTCCGGTCAG  R: AGCTGGCTTTTCGTATATTGTG |
| Caspase1 | F: GCCTGTTCCTGTGATGTGGAG  R: TGCCCACAGACATTCATACAGTTTC |
| GAPDH | F: CTCCTTTTTGGCAGACACTGGTG  R: ATGCCAGTGAGCTTCCCGTTCAG |

*TXNIP*, thioredoxin interacting protein; *XIAP*, X-linked inhibitor of apoptosis protein; *BIRC3*, baculoviral IAP repeat containing 3; *CBLB*, Cbl-b ubiquitin ligases; *RBX1*, ring-box protein 1; *NLRP3*, nod-like receptor pyrin domain containing protein 3; *ASC,* associated speck-like protein containing CARD.
